# Population genetic structure of the malaria vector *Anopheles minimus* in Thailand based on mitochondrial DNA markers

**DOI:** 10.1186/s13071-021-04998-7

**Published:** 2021-09-26

**Authors:** Kamonchanok Bunmee, Urusa Thaenkham, Naowarat Saralamba, Alongkot Ponlawat, Daibin Zhong, Liwang Cui, Jetsumon Sattabongkot, Patchara Sriwichai

**Affiliations:** 1 Department of Medical Entomology, Faculty of Tropical Medicine, Mahidol University, Bangkok, Thailand; 2 Department of Helminthology, Faculty of Tropical Medicine, Mahidol University, Bangkok, Thailand; 3 Department of Molecular Tropical Medicine and Genetics, Faculty of Tropical Medicine, Mahidol University, Bangkok, Thailand; 4 Department of Entomology, Armed Forces Research Institute of Medical Sciences (AFRIMS), Bangkok, Thailand; 5 Program in Public Health, University of California at Irvine, Irvine, CA 92697 USA; 6 Division of Infectious Diseases, Department of Internal Medicine, Morsani College of Medicine, University of South Florida, Tampa, FL 33612 USA; 7 Mahidol Vivax Research Unit, Faculty of Tropical Medicine, Mahidol University, Bangkok, Thailand

**Keywords:** Malaria vector, *Anopheles minimus* lineages A and B, Population genetic structure, Mitochondrial protein-coding genes, Thailand

## Abstract

**Background:**

The malaria vector *Anopheles minimus* has been influenced by external stresses affecting the survival rate and vectorial capacity of the population. Since *An. minimus* habitats have continuously undergone ecological changes, this study aimed to determine the population genetic structure and the potential gene flow among the *An. minimus* populations in Thailand.

**Methods:**

*Anopheles minimus* was collected from five malaria transmission areas in Thailand using Centers for Disease Control and Prevention (CDC) light traps. Seventy-nine females from those populations were used as representative samples. The partial mitochondrial cytochrome *c* oxidase subunit I (*COI*), cytochrome *c* oxidase subunit II (*COII*) and cytochrome b (*Cytb*) gene sequences were amplified and analyzed to identify species and determine the current population genetic structure. For the past population, we determined the population genetic structure from the 60 deposited *COII* sequences in GenBank of *An. minimus* collected from Thailand 20 years ago.

**Results:**

The current populations of *An. minimus* were genetically divided into two lineages, A and B. Lineage A has high haplotype diversity under gene flow similar to the population in the past. Neutrality tests suggested population expansion of *An. minimus*, with the detection of abundant rare mutations in all populations, which tend to arise from negative selection.

**Conclusions:**

This study revealed that the population genetic structure of *An. minimus* lineage A was similar between the past and present populations, indicating high adaptability of the species. There was substantial gene flow between the eastern and western *An. minimus* populations without detection of significant gene flow barriers.

**Graphical abstract:**

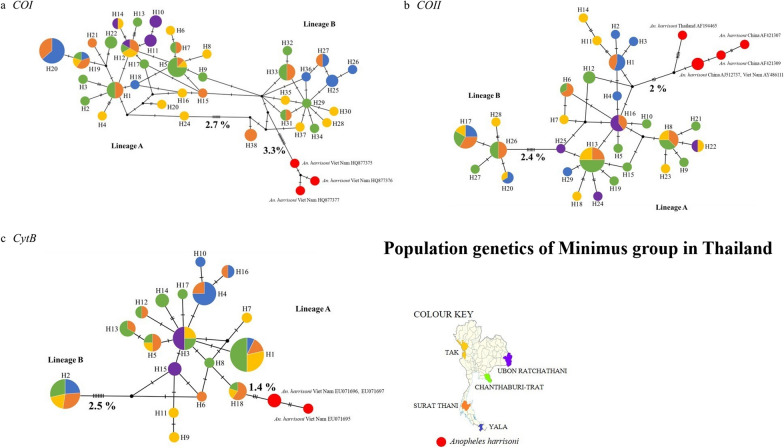

**Supplementary Information:**

The online version contains supplementary material available at 10.1186/s13071-021-04998-7.

## Background

*Anopheles minimus* is one of the principal malaria vectors in Southeast Asia, including Thailand [1]. The minimus group complex contains three species, *An. minimus* lineage A, *An. harrisoni* and *An. yaeyamaensis* [2]. In Thailand, species complex A is the dominant type, and is distributed throughout the country [2]. This species is the most commonly found in areas of high malaria transmission [1–4]. *Anopheles minimus* has an anthropophilic preference and is an important vector of indoor malaria transmission [3, 5]. External environmental stress impacts the vector survival rate, behavior, ecology, vectorial capacity, and host–pathogen interactions. External stress, consisting of climate change, changes in land use, host migration, and insecticide use, plays a vital role in selective pressure on mosquito populations [6]. These environmental factors can induce changes in surface temperature and ecosystem balance, contributing to vector development, evolution, reproductive isolation among populations, and increases in disease transmission [6, 7]. Such climate change affecting the natural environment drives the evolution of both vector and host [8].

The local adaptation of *An. minimus* in Vietnam appears to have been affected by genetic differentiation between populations, forced by ecotypic selection based on intraspecific behavioral differences and ecology in specific habitats [9]. The partial analysis of the *COI* gene sequence has been used to assess historical and current gene flow among *An. albimanus* populations in the Caribbean and the Pacific regions of Colombia, which showed high genetic differentiation influenced by specific ecological conditions, human migration, and activity [10]. Chen et al. investigated gene flow among populations of *An. minimus* in Southeast Asia using an analysis of partial mitochondrial cytochrome *c* oxidase subunit II (*COII*) [11]. Reconstruction of the phylogenetic relationships of *An. minimus* revealed that two different lineages, A and B, coexist in malaria transmission areas. These two lineages may split and expand to facilitate adaptation under different eco-climatic conditions. A previous study has shown that lineage A spread throughout Thailand, while lineage B was restricted to certain areas [11].

The mitochondrial protein genes are appropriate molecular markers to determine the population genetic structures of species. Mitochondrial DNA (mtDNA) sequence data have been used to investigate the genetic variations and phylogenetic relationships of different species and to accurately assess gene flow and differences among populations [12–17]. Several mtDNA sequences are sensitive to genetic drift and are particularly useful for analyzing the genetic diversity and genetic structure of populations. Variations in mtDNA can reflect the demographic history of invading mosquitoes [18]. Further, the mtDNA markers have been widely used in the studies of the population genetic structure of mosquitoes, including *An. minimus* [11], *An. sinensis* [19], *An. baimaii* [20], *An. dirus* [21–23], *An. lesteri* [24], *An. darling* [25], *An. stephensi* [26], *Ae. aegypti* [27, 28], and *Ae. albopictus* [29, 30].

Populations experience different selective pressures due to changing environmental factors and human behavior. The mitochondrial protein-coding genes were used to investigate whether there has been any differentiation of the genetic structure of *An. minimus* in Thailand in the past 20 years. Therefore, the aims of this focused population genetic study were (i) to determine the current population genetic structure and the potential gene flow by investigating genetic differentiation among the populations of *An. minimus* and (ii) to compare the population genetic structure of between the current and past *An. minimus* population of 20 years ago. The understanding of the population genetic structure and the potential gene flow among the populations gained from this study can be used as molecular tools for monitoring mosquito populations and developing guidelines for malaria vector control strategies in Thailand. Such information can also be used to measure and monitor gene flow or spread of vector populations after applying a control measure.

## Methods

### Mosquito collection and morphological identification

**Fig. 1:**
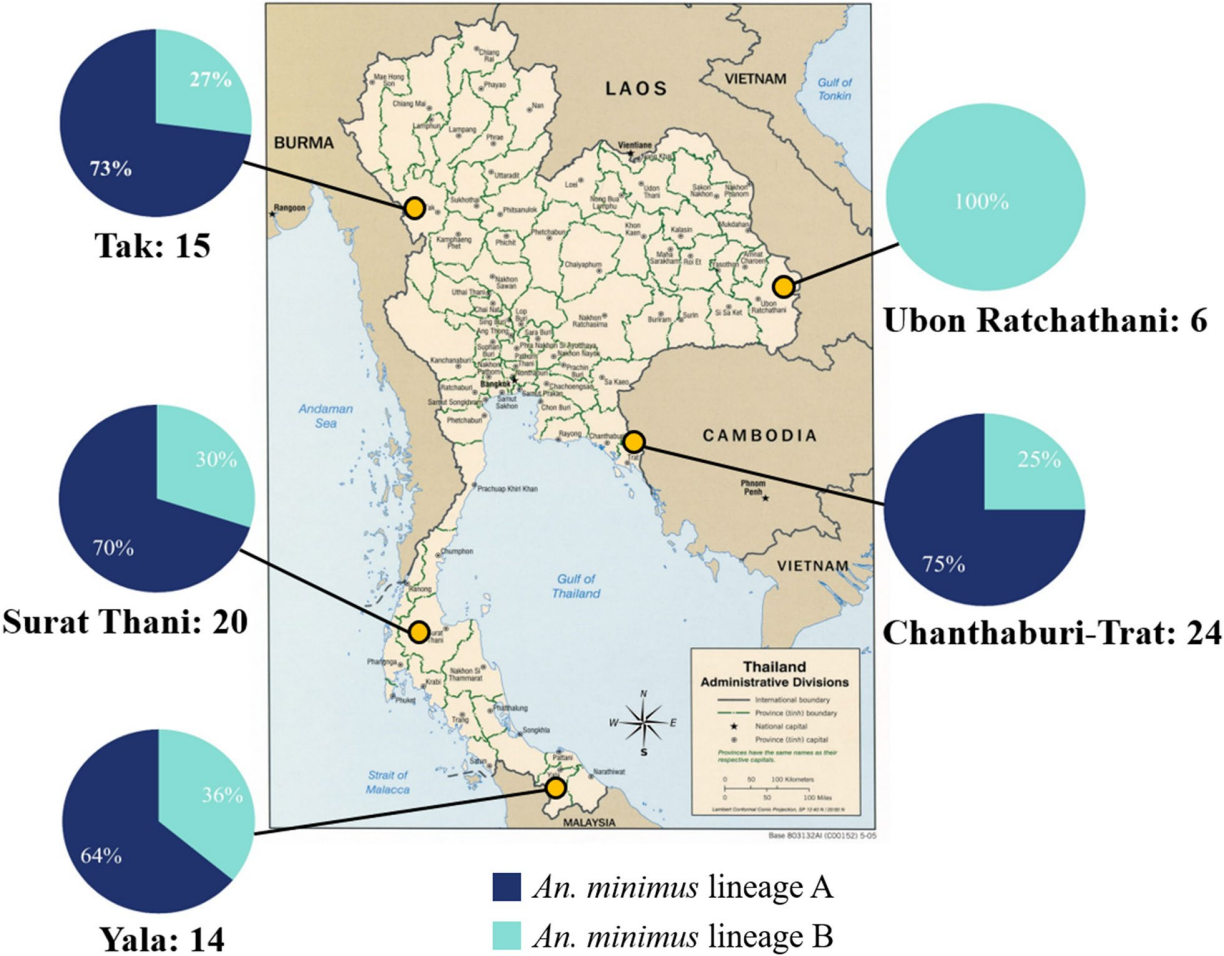
Map of the mosquito collection sites in the malaria transmission area from six provinces throughout Thailand: Tak, *n* = 15; Surat Thani, *n* = 20; Yala, *n* = 14; Ubon Ratchathani, *n* = 6; and Chanthaburi-Trat, *n* = 24. *Anopheles minimus* lineage A and B distributions overlap in each collection site (lineage A, blue color; lineage B, green color)

A total of 79 adult female *An. minimus* were collected from five different populations in malaria transmission areas along the western and eastern borders of Thailand, including Tak, Surat Thani, Yala, Ubon Ratchathani, and Chanthaburi-Trat provinces. The collection sites were selected according to the malaria operational plan report FY2018 [31] (Fig. 1), which was based on the identification of malaria transmission areas and malaria vector distribution areas. Adult mosquitoes were collected between 2016 and 2019 using the Centers for Disease Control and Prevention miniature light traps (CDC-LT). Twenty CDC-LTs were randomly placed around each village. The traps were placed about 50 m from each other in 10 selected houses for four consecutive nights. The mosquito specimens were stored individually in 1.5-ml microcentrifuge tubes at −20 °C. They were then morphologically identified based on the taxonomic keys of mosquitoes in Thailand [32]. All specimens identified as *An. minimus* sensu lato were selected and further used for molecular identification to confirm the species identification.

### Molecular identification

Genomic DNA of *An. minimus* was extracted from the abdomens of the mosquitoes using the Genomic Mini Kits (Geneaid Biotech Ltd., Taipei, Taiwan) following the manufacturer's instructions, except for the change of the lysis period to overnight at 60 °C. The partial mitochondrial genes *COI*, *COII*, and *Cytb* were used as genetic markers for molecular identification. The primers were designed based on the *An. minimus* complete mitochondrial genome (GenBank: KT895423) and gene-specific sequences with the most informative regions of each gene (Additional file 1: Table S1). The primers for polymerase chain reaction (PCR) were analyzed for suitability using OligoCalc, an online oligonucleotide properties calculator [33].

The PCR amplifications were performed in a final volume of 20 µl containing genomic DNA, 1× OnePCR™ PCR reaction mixture (GeneDireX, Inc., Taiwan), and 10 pmol of forward and reverse primers for each genetic marker (Additional file 2: Table S2). PCR consisted of initial denaturation at 95 °C for 3 min, followed by 35 cycles of denaturation at 95 °C for 45 s, annealing at 52 °C (for primers of *COII* and *Cytb*) and 54 °C (for primers of *COI)* for 40 s and extension at 72 °C for 50 s, and a final extension at 72 °C for 8 min. The PCR products were subsequently purified using Gel/PCR DNA fragments extraction kits (Geneaid Biotech Ltd., Taipei, Taiwan). The purified PCR products were sequenced with the same primers as conventional PCR by Sanger sequencing using a 3730xl analyzer (Thermo Scientific, USA), serviced by Bio Basic, Inc., Singapore.

### Phylogenetic analyses

A phylogenetic tree for *An. minimus* from the five different populations was constructed with *An. harrisoni* (*An. minimus* complex C) using the maximum likelihood (ML) method implemented in the MEGA 7 program [34]. The ML was performed using a best-fit nucleotide substitution model with 1000 bootstrap replications for tree topology support. The nucleotide sequences of *An. harrisoni* were the results of previous studies retrieved from the GenBank database with accession numbers HQ877375–HQ877377 (*COI*), AF421307, AF421309, AY486111, AF417707, KT899887, KF687432 (*COII*), and EU071695–EU071697 (*Cytb*) [35–40]. *Anopheles dirus* was used as an outgroup with accession numbers AF417707 (*COI*), KT899887 (*COII*), and KF687432 (*Cytb*) [39–41]. The GenBank accession numbers MT651216–MT651294 (*COI*), MT663561–MT663639 (*COII*), and MT663640–MT663718 (*Cytb*) were also included as representative sequences of lineage A and lineage B (Additional file 3: Table S3, Additional file 4: Table S4, Additional file 5: Table S5).

### Population genetic structure analyses

To determine the genetic diversity within the *An. minimus* population, haplotype diversity (Hd) and nucleotide diversity (*π*) were calculated using the DnaSP program, version 6 [42]. The haplotype relationships were estimated using a median-joining (MJ) network under pairwise nucleotide difference between haplotypes in the PopART 1.7 program [43].

Pairwise *F*-statistics (*F*_ST_) were computed based on the variance in allele frequencies to detect genetic differentiation among the populations using the ARLEQUIN 3.5.1.2 program [44]. The level of gene flow among the populations was estimated by measuring the numbers of migrants in a population per generation (*N*m) using the *F*_ST_ variances. Analysis of molecular variance (AMOVA) was conducted among the different geographical populations using ARLEQUIN to calculate the proportion of genetic variation within and between populations.

### Neutrality and demographic history

The frequency distribution of pairwise nucleotides was different between the observed and the expected distribution (mismatch distribution) under the expansion model of population demography implemented in ARLEQUIN [44] to examine historical demographic expansions. The sum of squared deviation (*SSD*) between the observed and expected mismatch distribution was performed as a test statistic to reflect a significant *SSD* (*P* < 0.05) value of historical demographic population expansion or contraction. Historical demographic expansions were also determined by neutrality tests conducted using two approaches, Tajima’s *D* [45] and Fu's *F*s [46] tests, related to natural selection.

### Changes in genetic structure of *An. minimus* population in Thailand over the past 20 years

To compare the population genetic structure of *An. minimus* between the present and 20 years ago, 60 sequences of *An. minimus* populations in Thailand were investigated using population genetic structure analysis, tests of neutrality, and demographic history events using ARLEQUIN, using the *COII* gene from the GenBank database accession numbers FN433526–FN433595 (Additional file 6: Table S6) [11].

Secondary data, including mean surface temperature data sets from 2000 to 2020 [47] and historical data about land-use changes and the forestry sector in Thailand from 2001 to 2018 [48], were analyzed to determine the fluctuation of environmental factors over the past two decades, which may have influenced changes in population structure.

## Results

### Mosquito collection and identification

A total of 79 *An. minimus* s.l. were collected from five populations from Tak (*n* = 15), Surat Thani (*n* = 20), Yala (*n* = 14), Chanthaburi-Trat (*n* = 24), and Ubon Ratchathani (*n* = 6) (Fig. 1). All specimens were morphologically identified as *An. minimus* s.l. before subjecting to molecular identification.

### Phylogenetic relationships of *An. minimus*

**Fig. 2:**
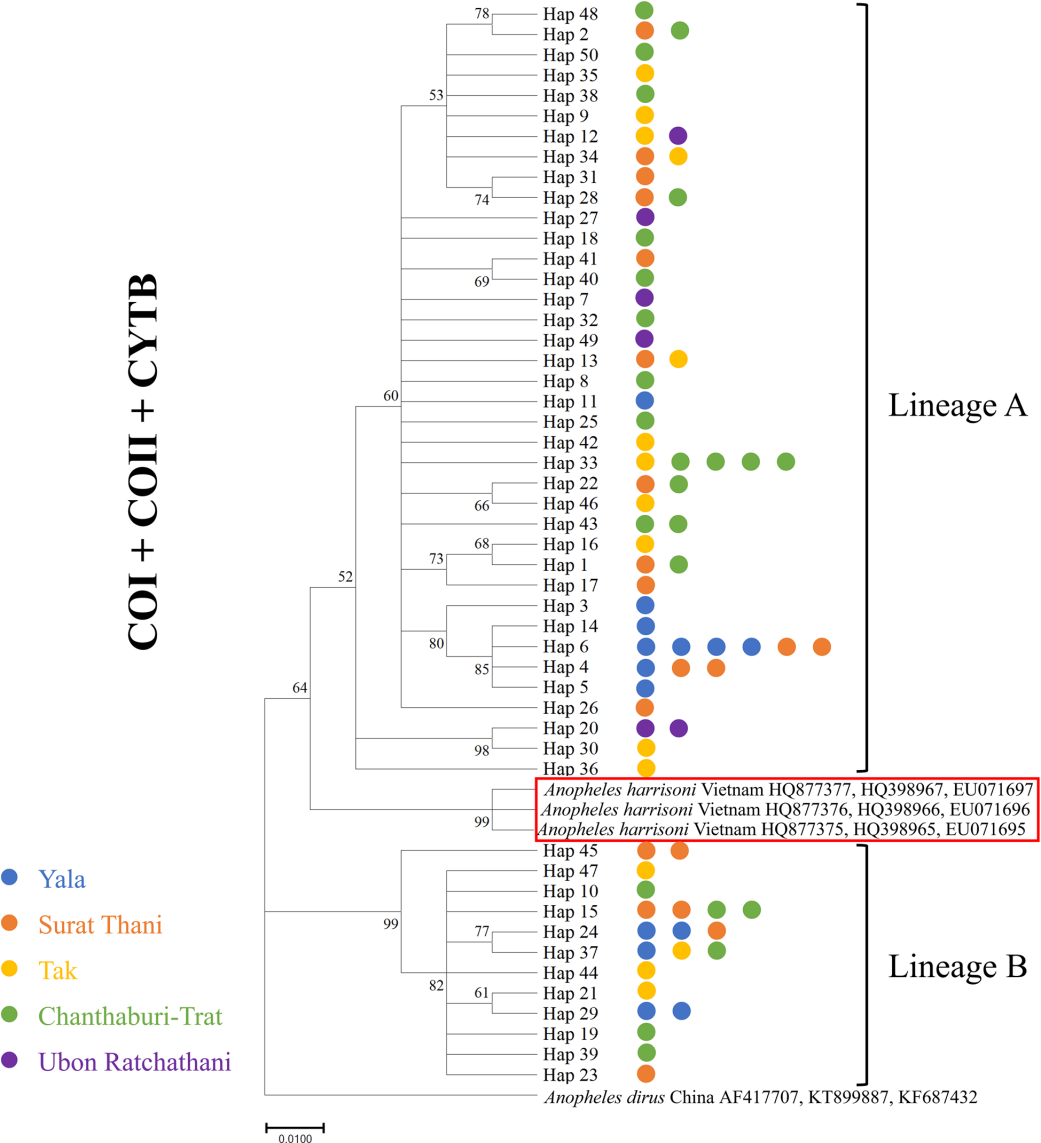
The maximum likelihood (ML) tree of two different lineages of *An. minimus*. The *An. harrisoni* is in the red circle based on analysis of the concatenated sequences of the mtDNA *COI*, *COII,* and *Cytb* genes with *An. dirus* used as outgroup. The labels in the tree include haplotype codes, and color depicts the different populations

**Fig. 3:**
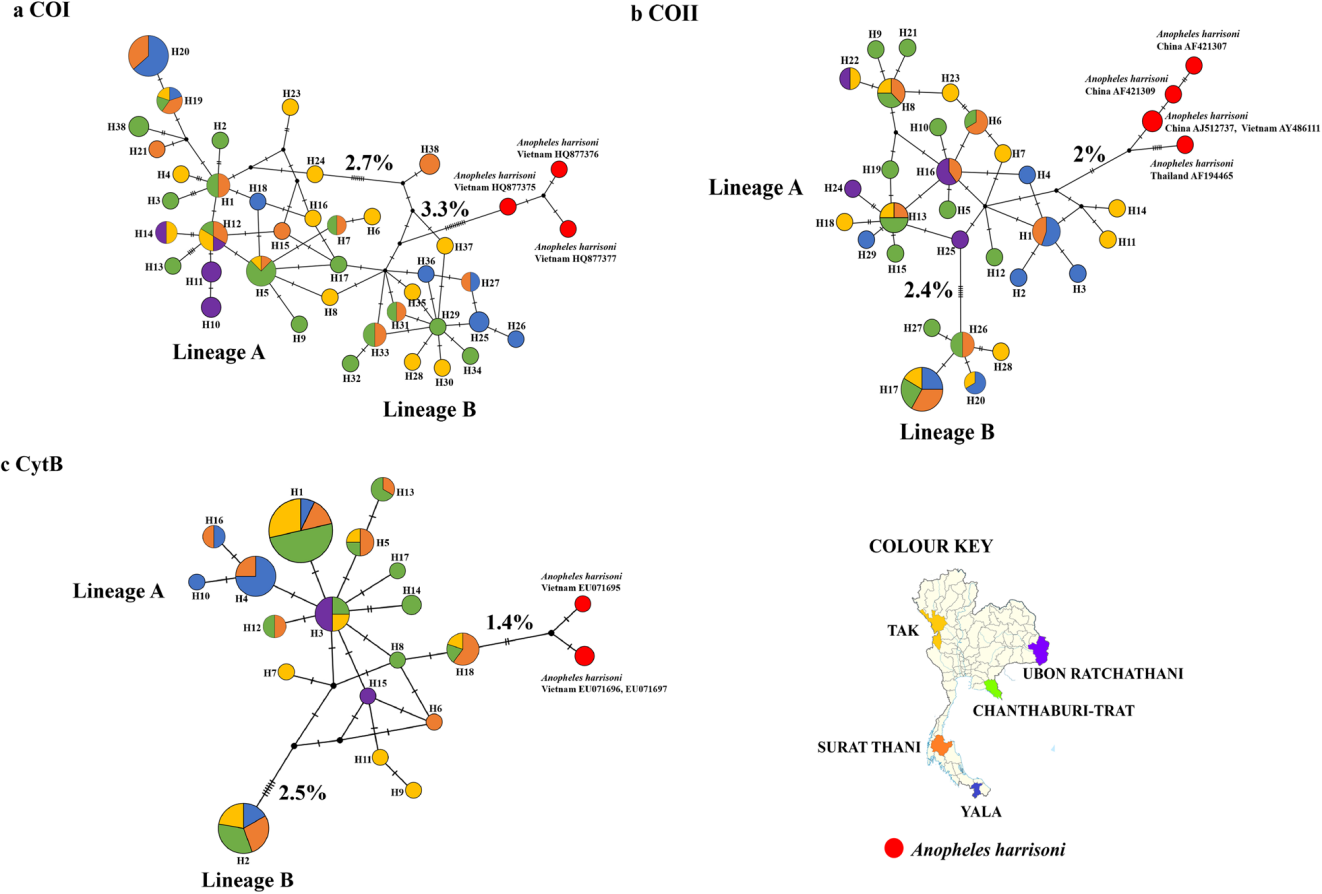
Median-joining haplotype network generated using PopART 1.7 for the *An. minimus* population corresponding to their geographical distribution separated into five populations in Thailand. **a** Haplotype network of the *COI* gene. **b** Haplotype network of *COII*. **c** Haplotype network of *Cytb*. Color represents different populations: Tak = yellow; Surat Thani = orange; Yala = blue; Ubon Ratchathani = purple; and Chanthaburi-Trat = green. Each haplotype is represented by a circle in which the circle size is proportional to the haplotype frequency. Mutations between haplotypes are indicated by lines representing mutations from the common haplotype. The red color represents the *An. harrisoni* population

We produced an alignment of 1330 bp of concatenated mitochondrial protein-coding sequences from 79 specimens. All mosquitoes were identified as *An. minimus* complex A, which belongs to the Minimus complex. The ML phylogenetic relationships indicated the presence of two genetically distinct lineages, A and B, in the current *An. minimus* population. Lineage A was the predominant population, with 58 individuals, while the other 21 individuals were of lineage B (Fig. 2). The ML phylogenetic relationship of each gene is shown in (Additional file 7: Figure S1). The average pairwise sequence divergence between *An. minimus* lineages A and B revealed 2.4–2.7% difference (Fig. 3). Although the populations of both lineages were distributed in the same area, it was found that the genetic distance within the population of lineage A (0.8%) was higher than that of lineage B (0.3%). *An. minimus* lineage A was genetically closer to *An. harrisoni* (1.4–2%) than *An. minimus* lineage B (Fig. 3).

### Genetic diversity

The sampled population had a high level of genetic diversity, with the numbers of haplotypes within the population ranging from five (Ubon Ratchathani) to 14 (Chanthaburi-Trat) haplotypes of lineage A and four to five haplotypes of lineage B (Additional file 8: Table S7). The overall haplotype diversity (Hd) level of lineages A and B was high, 0.97822 and 0.96190, respectively, with low nucleotide diversity (*π*) of 0.00639 and at least 0.00295, respectively. The haplotype diversity of each population ranged from Hd = 0.8 to 1 (Additional file 8: Table S7). The nucleotide diversity of lineage B (*π* = 0.002 to 0.003) was lower than that of lineage A (*π* = 0.003 to 0.008) (Additional file 8: Table S7).

The relationships among the *An. minimus* haplotypes were drawn using MJ, visualizing the haplotype frequencies and mutational steps among the haplotypes. Each haplotype is represented by a circle in which the circle size is proportional to the haplotype frequency. Mutations between haplotypes are indicated by lines representing mutations from the common haplotype. The haplotype network structure has a star-like phylogeny, with the most relevant single haplotypes surrounding the common haplotype. Lineage A was found in all locations, whereas lineage B did not occur in Ubon Ratchathani province (Fig. 3).

### Population genetic structure

Genetic differentiation among *An. minimus* populations was estimated using *F*_ST_ pairwise comparison (Additional file 9: Table S8). The Ubon Ratchathani population was the most divergent population of lineage A, with differentiation among the other populations ranging from 0.06657 to 0.58954 as present data on the pairwise *F*_ST_ values (below diagonal). The level of genetic differentiation of the Chanthaburi-Trat population was also divergent from other populations of lineage B (above diagonal) (Additional file 9: Table S8). The overall genetic differentiation based on the *F*_ST_ value was significantly low among the populations, ranging from 0.09–0.18 (*P* < 0.05). The high level of migration (*N*m) in the range of 1 to 2.5 was estimated from *F*_ST_ variances, indicating considerable gene flow among the populations (Table 1).

**Table 1:** Neutrality test, sum of squared deviation (*SSD*), and analysis of molecular variance (AMOVA) of the concatenated mitochondrial genetic markers in the *An. minimus* populations

Overall	Lineage A	Lineage B
Neutrality test		
Tajima’s *D*	−1.31	−1.18
Fu’s *F*s	−21.50*	−8.09*
*SSD*	0.004	0.02
Western and Eastern		
Percentage of variation		
Among groups	2.14	−4.11
Among populations	16.23*	12.72
Within populations	81.63*	91.39
Fixation indices		
*F*_ST_	0.18375*	0.08612*
*N*m	1.11054	2.65293
Regions		
Percentage of variation		
Among groups	5.74	−7.34
Among populations	12.83*	16.42
Within populations	81.44*	90.93
Fixation indices		
*F*_ST_	0.18563*	0.09072*
*N*m	1.09677	2.50573

AMOVA, *P < 0.05

*SSD*, sum of squared deviation; *F*_ST_, genetic differentiation; *N*m, number of migrations

Based on the geographical distribution of the *An. minimus* populations, AMOVA was conducted on different groupings (Table 1). The genetic differentiation indices (*F*_ST_) of both lineages A and B were determined for the populations in different sides of the country (the western and eastern parts) and by geographic regions (north, south, and east). These populations were significantly different, with a low value of *F*_ST_. A high level of migration (*N*m > 1) of *An. minimus* between populations was also measured by the *F*_ST_ indices (Table 1). A high level of genetic variation of more than 81% was detected within the population, whereas a low level of genetic variation (12–16%) was observed between the populations (Table 1).

We hypothesized that the populations observed in this study had a genetic structure that arose by gene flow in the main population of *An. minimus* lineage A. Nonsignificant genetic variation within and among the populations was detected in lineage B. This evidence indicated that these populations of *An. minimus* lineage B had no differentiation between individuals collected from geographical isolation.

### Selection and demographic history

**Fig. 4:**
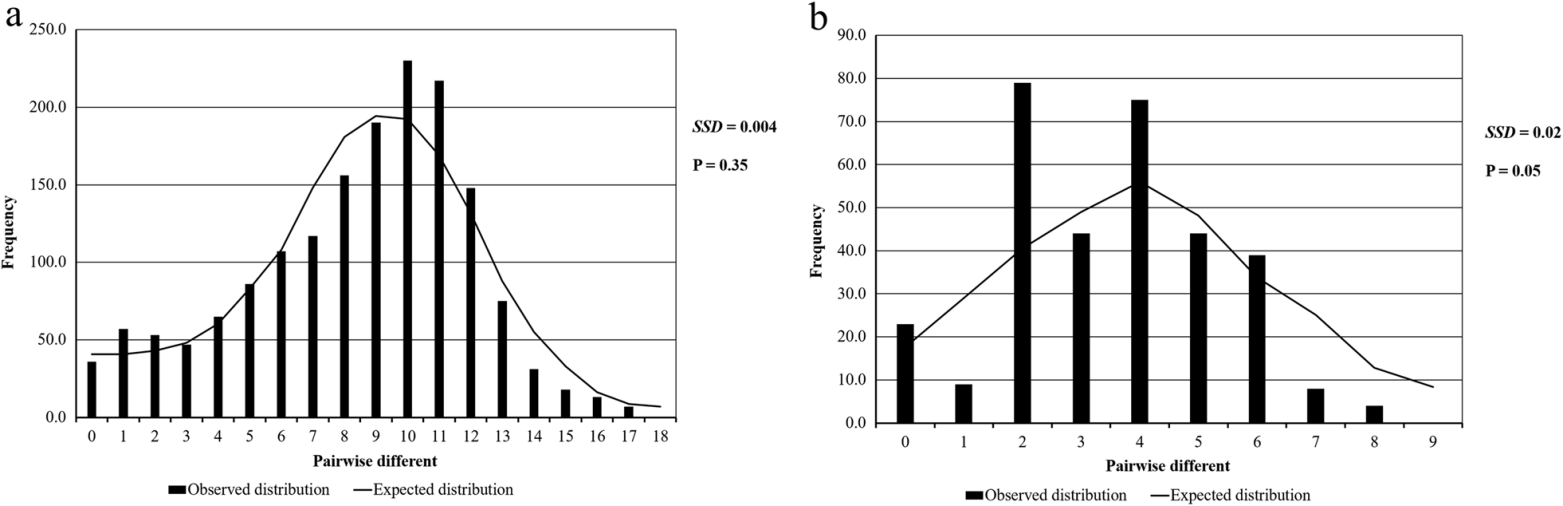
Mismatch distribution of the *An. minimus* population according to two distinct lineages. **a** Mismatch distribution of lineage A. **b** Mismatch distribution of lineage B. Bar represents the observed distribution of pairwise differences, whereas the line shows the expected distribution under the sudden expansion model

As depicted in Fig. 4, historical demographic expansions of populations produce a characteristic smooth unimodal or bell-shaped pattern of population expansion. The hypothesis of sudden expansion indicated by mismatch distribution analysis and the *SSD* was not significant for all populations (Table 2). A nonsignificant *SSD* *P*-value (*P* < 0.05) indicated that the goodness of fit of the observed and expected mismatch distribution models is likely to be the same, leading to the acceptance of the population expansion model. Tajima’s *D* tests were not statistically significant for negative *D* values (Table 2), whereas Fu’s *F*s tests were significantly negative for *F*_*S*_ values (*P* < 0.05).

**Table 2:** Analysis of molecular variance (AMOVA), fixation indices (*F*_ST_), and neutrality test comparison between the historical and recent populations of *An. minimus* in Thailand

Statistical analysis	Current populations		Previous populations [11]	
	Lineage A	Lineage B	Lineage A	Lineage B
Percent variation				
Among group	5.96253	−1.56655	−1.29	15.95
Among populations	7.8791	−3.49905	23.17	0.58
Within population	86.16*	105.06	78.12	83.47
Fixation indices				
*F*_ST_	0.13842 *	−0.05066	0.21877 *	0.1653
*N*m	1.5561	−5.18486	0.89275	1.2624
Neutrality test				
Tajima’s *D*	−1.16786	−0.72139	−1.35968	−0.8232
Fu’s *F*s	−15.49214 *	−0.90891	−11.30319 *	−1.75379 *
Mismatch distribution analysis				
*SSD*	0.006341	0.010402	0.005227	0.011001

AMOVA, *P < 0.05

*SSD*, sum square deviation; *F*_ST_, genetic differentiation; *N*m, number of migrations

### Comparison of genetic structure of *An. minimus* populations in Thailand over the past 20 years

According to the analysis of the *COII* gene, the *An. minimus* population genetically separated into lineages A and B in the course of the past 20 years. The comparison of the genetic distances between the populations of lineages A and B had similar patterns; the distance was 2.2% in the past population and 2.4% in the current population. Analysis of the genetic diversity of the entire population in previous studies compared to the current population indicated that the level of haplotype diversity remained the same, with high haplotype diversity in the range Hd = 0.6–1 in every population. However, the nucleotide diversity was low (*π* = 0 to 0.01).

The comparison of population genetic structure between the present and 20 years ago showed that the number of migrations, representing gene flow among the population, has increased over the past 20 years. Population expansion under negative selection was detected by the strong negative *D* and *F*s neutrality test values, and the lack of significance between the observed and expected distribution of mismatch analysis (Table 2).

## Discussion

In this study, we describe the genetic structure of *An. minimus* populations in Thailand, which is essential for future management of malaria vectors [18, 49]. The population genetic structure was analyzed based on mtDNA gene sequences, which provided information about the genetic diversity, genetic differentiation, gene flow, and selection within and between populations. This study identified a large genetic distance indicating significant divergence between the two lineages of *An. minimus*.

The *An. minimus* population has been undergoing high recombination across its geographic distribution, reducing genetic variation and increasing the number of closely related haplotypes. Haplotype sharing existed between the populations, even those that are separated by a large geographic distance. Moderate *F*_ST_ values were obtained from pairwise comparisons among the populations within the different geographical distribution zones, with much more genetic variation within the population than between the populations. This observation suggested that gene flow occurred among recent *An. minimus* populations without geographic barriers throughout Thailand. Hence, demographic population expansion under negative selection was observed. There was an excess of rare mutations in all populations, reducing genetic variation within populations due to gene flow.

However, the comparison of population genetic structures between the past and the current populations of *An. minimus* in Thailand revealed a persistent genetic structure with a similar pattern of moderate genetic differentiation, population expansion, and negative selection. It may indicate that the *An. minimus* populations had free genetic exchange among the populations, as evaluated from the reduced genetic variance and genetic differentiation among populations.

The mtDNA is a suitable genetic marker and has been extensively used in evolutionary studies. The *Anopheles* mosquito mtDNA genes have been used to evaluate the genetic structure of mosquito populations [18–20, 40, 50]. MtDNA offers many advantages. First, the uniparental mode of maternal inheritance is specific in sexually isolated demes or lineages. Second, the MtDNA has a 5–10 times higher evolutionary rate than nuclear DNA, and is therefore widely used to determine gene frequencies and the effects of natural selection on intraspecific genetic variation. Finally, it lacks normal recombination, therefore reflecting only a single genealogical history of each genome [51, 52]. Using the mtDNA sequence data as a genetic marker allowed the measurement of genetic differentiation within and between the populations, producing evidence of gene flow and population expansion without limitation by geographic distance.

To reduce bias in marker selection, a combination of several mtDNA gene sequences was used to describe the overall population structure. Assessment of the population genetic structure using single-gene and multi-gene concatenated markers found that these populations present the same population structure, as follows. (1) The recent *An. minimus* population has high gene flow between the populations. (2) The population tends to undergo demographic expansion with no constraints from a geographic distance or geographical barriers. (3) These populations experienced exposure to negative selective pressure. The negative selection maintained their genetic structure by removing deleterious mutations [53]. Hence, the multi-gene concatenated markers were also used to increase the assessment accuracy of genetic relationships and population structure [54, 55].

Mitochondrial protein-coding genes have been used to evaluate the selective pressure acting on mosquitoes’ mitogenomes [40]. Several studies on malaria vector mosquitoes, including *An. subpictus*, *An. peditaneatus*, and *An. vagus* from five different localities of Sri Lanka [18], *An. sinensis* in China [19], *An. baimaii* in northeast India [20], *An. dirus* in Southeast Asia and China [21–23], and *An. minimus* across China, Thailand, and Vietnam [11], have been conducted to analyze the genetic diversity and population genetic structure, using the mtDNA genes as the genetic marker.

The coexistence of *An. minimus* lineages A and B in the current population was observed in the same habitat of the active transmission area. However, it is not clear whether they differ in vectorial capacity for malaria transmission. The dominant lineage in the *An. minimus* populations was lineage A. Therefore, the abilities for genetic exchange between *An. minimus* lineages A and B were considered, with the noticeable genetic differences between the two lineages and the potential for gene flow among the populations evaluated separately. This study identified the distribution of both lineages in western and eastern Thailand. Earlier studies found lineage B only in western Thailand and suggested expansion through India [11, 56].

A similar, low to moderate genetic differentiation level was observed in both lineages A and B, indicating possible gene flow between the populations in both lineages. No differences were observed in the pairwise comparison of the separated groups in the western and eastern regions, despite separation by land-use and urbanization changes. The low levels of genetic differentiation between the western and eastern populations suggest the lack of significant geographical barriers limiting gene flow. Alternatively, these populations might have exchanged genetic materials before being separated by urban areas as barriers, while gene flow between these populations could have been assisted by human activities. In previous studies, gene flow among malaria vectors was observed due to the absence of geographic distance and geographical barriers [18, 57–60]. It has been suggested that geographical barriers are the main mechanism of maintenance of gene flow between the populations [18].

Demographic inference tests (Tajima’s *D* and Fu’s *F*s) [61] revealed that the most recent population of *An. minimus* experienced a population expansion. Population expansion was also suggested by the relatively high haplotype diversity and low nucleotide diversity observed, which indicated that the recent populations diverged from each other by rapid demographic expansion [62–64]. All statistical tests of neutrality reported negative values, indicating an excess of low-frequency mutations due to the evolutionary forces operating on the populations. The demographic history of malaria vector populations in Southeast Asia also indicated that *An. dirus* and *An. aconitus* populations in Southeast Asia experienced population expansion under significant negative selection [22, 65].

The *An. minimus* populations of 20 years ago had high genetic diversity, and we found evidence of population expansion. The mosquito populations in Southeast Asia are also affected by human activities such as deforestation and vector control, shaping the distribution and genetic variation within species [11]. Hence, this comparative study of population genetic structure between the present and 20 years previous populations found results inconsistent with those of the previous report [11]. External selective pressure appears to have impacted the population genetic structure. We found that the population genetic structure of *An. minimus* in Thailand was similar to the structure detected 20 years ago, despite the changes in environmental factors over time [47, 48]. For this reason, evidence of population expansion and gene flow among the populations might be necessary to explain the adaptative behavior for sustaining the vector capacity and transmitting malaria. Some of the mutations associated with the vector abilities, transmission, and insecticide resistance could be transferred to other populations, affecting the distribution of malaria [66].

Mosquito populations can rapidly adapt in response to changes in environmental conditions, such as climate change and human activities, which might influence the mosquito's survival rate, population density, and ecological distribution [67]. Therefore, environmental factors play an essential role in the evolutionary process, resulting in changes in factors such as the mosquito's interactions with the environment, genetic diversity within species at the population level, and gene flow (Additional file 10: Figure S2) [68–70]. Our results indicate that the presence of gene flow between *An. minimus* populations in Thailand might be impacted by environmental factors, enhancing gene flow among the populations studied, consistent with previous studies [68–72]. The average annual surface temperature in Thailand has increased by approximately 1 °C over the past 20 years (2000 to 2019) (Additional file 11: Figure S3) [47, 73]. The temperature change has also caused a decrease in the frequency of tropical cyclones entering Thailand, resulting in significant changes in rainfall patterns [74]. The increasing surface temperature could have enhanced the reproductive rates of mosquitos [75–77]. These factors also restricted the species or occurrence of sympatric populations, forcing the vector to migrate to better habitats.

Human activities and land-use change could have driven intraspecific divergence [78]. According to the land-use change data based on tree cover loss in Thailand from 2001 to 2018, natural forest areas were replaced by plantation or commercial agricultural areas (Additional file 11: Figure S3) [48]. These land-use changes, such as new plantation areas or rubber plantations, provide an ideal habitat for *Anopheles* spp., potentially leading to increases in the vector density and the re-emergence of *An. minimus* [79]. Similarly, the genetic differentiation and gene flow between *An. funestus* populations have been shaped by various factors, not only geographical distance, but also the consequence of different breeding sites, mosquito migration, environmental changes, and human activities [80]. Finally, insecticide use can impact genetic diversity due to population migration, leading to genetic exchange between populations [81–83].

The analysis of genetic differentiation between the populations in lineage B is still incomplete. There is little information on vector biology between the different genetic lineages of *An. minimus*. This study showed that lineage B had a population structure similar to lineage A. This study has limitations in collecting representative populations in eastern Thailand. Further studies are required to increase the sample sizes, and seasonal sampling design by generations may provide accurate results about the specific rate of population growth and dynamics. This study investigated the demographic history of population expansion, but cannot specify the direction and rate of population growth, a topic worthy of attention in future studies.

## Conclusions

This study identified the coexistence of two lineages of *An. minimus* in both eastern and western Thailand. Gene flow was apparent among the geographically distant *An. minimus* populations, with no evidence of impact by external selective pressure, environmental changes, and geographical barriers. In addition, the population genetic structure of *An. minimus* populations was persistent in the past 20 years. Such information may be useful for developing and implementing local malaria vector control strategies and monitoring population spread in the face of control interventions.

## Supplementary Information

**Additional file 1: Table S1.** GenBank accession numbers as used for primer design.

**Additional file 2: Table S2.** Primer list of mitochondrial genetic markers used in this study.

**Additional file 3: Table S3.** The GenBank database accession numbers of cytochrome c oxidase subunit I (*COI*) gene.

**Additional file 4: Table S4.** The GenBank database accession numbers of cytochrome c oxidase subunit II (*COII*) gene.

**Additional file 5: Table S5.** The GenBank database accession numbers of cytochrome b (*Cytb*) gene.

**Additional file 6: Table S6.** The GenBank database accession numbers of cytochrome c oxidase subunit II (*COII*) gene as the 20 years *An. minimus* population sequences.

**Additional file 7: Figure S1.** The maximum likelihood (ML) tree of two different genetical lineages of *Anopheles minimus*, the *An. harrisoni* as in the red circle and the *An. dirus* used as outgroup. Bootstrap values less than 50% were excluded in the phylogenetic tree. **a** The ML tree of the *COI* gene. **b** The ML tree of the *COII* gene. **c** The ML tree of the *Cytb* gene. The labels in the tree include haplotype codes, and color indicates the different populations.

**Additional file 8: Table S7.** Number of individuals, number of haplotypes, haplotype diversity (*Hd*), nucleotide diversity (*π*).

**Additional file 9: Table S8.***F*_*ST*_ comparisons for all the five populations.

**Additional file 10: Figure S2.** Fluctuations in environmental factors enhance gene flow, including temperature change, land-use change, urbanization, deforestation, plantation, and insecticide use.

**Additional file 11: Figure S3.** Fluctuation in environmental factors from the past to the present. **a** Fluctuation of environmental factors (land-use change, mean surface temperature by day and night). **b** The average of mean daytime temperature from 2000 to 2019. **c** Average mean nighttime temperature from 2000 to 2019.

## Data Availability

The data sets used and/or analyzed during the current study are available from the corresponding author upon reasonable request. Representative sequences were submitted to the GenBank database with accession numbers MT651216–MT651294 (*COI*), MT663561–MT663639 (*COII*), and MT663640–MT663718 (*Cytb*).

## Abbreviations

*COI*: Cytochrome *c* oxidase subunit I
*COII*: Cytochrome *c* oxidase subunit II
*Cytb*: Cytochrome b
NJ: Neighbor-joining
*F*_ST_: Genetic differentiation
*N*m: Number of migrations
CDC-LT: Centers for Disease Control and Prevention miniature light traps
PCR: Polymerase chain reaction
ML: Maximum likelihood
Hd: Haplotype diversity
*π*: Nucleotide diversity
MJ: Median-joining
AMOVA: Analysis of molecular variance
*SSD*: Sum square deviation
*D*: Tajima’s *D* test
*F*s: Fu’s Fs test
s: Second
min: Minute
