# Supplementary material for: Population genetic structure of the malaria vector Anopheles minimus in Thailand based on mitochondrial DNA markers
Source: Parasit Vectors. 2021 Sep 26;14:496. doi: 10.1186/s13071-021-04998-7 (PMC8474755; doi:10.1186/s13071-021-04998-7)
Supplement: Supplementary file 1 — Additional file 1: Table S1. GenBank accession numbers as used for primer design. [file 13071_2021_4998_MOESM1_ESM.docx]

**Additional file 1: Table S1.** GenBank accession numbers as used for primer design

| Gene | GenBank accession number | Gene | GenBank accession number | Gene | GenBank accession number |
| --- | --- | --- | --- | --- | --- |
| *COI* | EF423577 | *COII* | AY486111 | *COII* | AY953362 |
| *COI* | EF423578 | *COII* | AY486110 | *COII* | AY953366 |
| *COI* | EF423583 | *COII* | HQ398965 | *COII* | AY953367 |
| *COI* | GQ259179 | *COII* | HQ398966 | *COII* | JQ042263 |
| *COI* | GQ259180 | *COII* | HQ398967 | *COII* | JQ042266 |
| *COI* | GQ259181 | *COII* | AF194466 | *COII* | JQ042269 |
| *COI* | GQ259187 | *COII* | AF194477 | *COII* | JQ042270 |
| *COI* | GQ259188 | *COII* | AF195048 | *COII* | KM373272 |
| *COI* | GQ259189 | *COII* | AF195049 | *COII* | KM373277 |
| *COI* | GQ906988 | *COII* | JQ042262 | *COII* | KM373279 |
| *COI* | GQ906989 | *COII* | JQ042264 | *COII* | KM373281 |
| *COI* | GQ906990 | *COII* | JQ042265 | *COII* | KM373284 |
| *COI* | GQ906991 | *COII* | JQ042267 | *COII* | KM373288 |
| *COI* | GQ906993 | *COII* | JQ042273 | *COII* | KM373290 |
| *COI* | GQ906995 | *COII* | JQ042274 | *COII* | KM373292 |
| *COI* | GQ906996 | *COII* | JQ042275 | *COII* | KM373296 |
| *COI* | GQ906998 | *COII* | JQ042276 | *COII* | FN433589 |
| *COI* | HQ398935 | *COII* | JQ042277 | *COII* | FN433591 |
| *COI* | HQ398936 | *COII* | JQ042278 | *COII* | FN433592 |
| *COI* | HQ398937 | *COII* | JQ042279 | *COII* | FN433593 |
| *COI* | HQ877375 | *COII* | JQ042283 | *COII* | FN433594 |
| *COI* | HQ877376 | *COII* | JQ042291 | *COII* | FN433595 |
| *COI* | HQ877377 | *COII* | KM373301 | *COII* | JQ046381 |
| *COI* | JN881335 | *COII* | KM373302 | *COII* | JQ046387 |
| *COI* | AY423057 | *COII* | KM373303 | *COII* | JQ046389 |
| *COII* | AF421306 | *COII* | AM396768 | *COII* | JQ046390 |
| *COII* | AF421308 | *COII* | AM396769 | *CytB* | KF382825 |
| *COII* | AF421309 | *COII* | AM396770 | *CytB* | KF431913 |
| *COII* | AJ512737 | *COII* | AF421305 | *CytB* | KF687398 |
| *COII* | AJ512738 | *COII* | AY953361 | *CytB* | JX070737 |
