# Supplementary material for: Population genetic structure of the malaria vector Anopheles minimus in Thailand based on mitochondrial DNA markers
Source: Parasit Vectors. 2021 Sep 26;14:496. doi: 10.1186/s13071-021-04998-7 (PMC8474755; doi:10.1186/s13071-021-04998-7)
Supplement: Supplementary file 2 — Additional file 2: Table S2. Primer list of mitochondrial genetic markers used in this study. [file 13071_2021_4998_MOESM2_ESM.docx]

**Additional file 2: Table S2.** Primer list of mitochondrial genetic markers.

| Genetic marker | Primer and sequence (5’ – 3’) | Amplicon size (bp) |
| --- | --- | --- |
| *COI* | COI_F CTGGAATTGCTCATGCTGGAG | 666 |
|  | COI_R CTCCTGTTAATCCTCCGACTG |  |
| *COII* | COII_F GGCAACATGAGCAAATTTAGG | 503 |
|  | COII_R GAAGGAACTGTTCAAGAATG |  |
| *CytB* | CYTB_F GTAGGTCGAGGAGTATATTATG | 499 |
|  | CYTB_R CAGGTTGAATATGAACTGGAG |  |
