# Supplementary figures and images for: Population genetic structure of the malaria vector Anopheles minimus in Thailand based on mitochondrial DNA markers
Source: Parasit Vectors. 2021 Sep 26;14:496. doi: 10.1186/s13071-021-04998-7 (PMC8474755; doi:10.1186/s13071-021-04998-7)

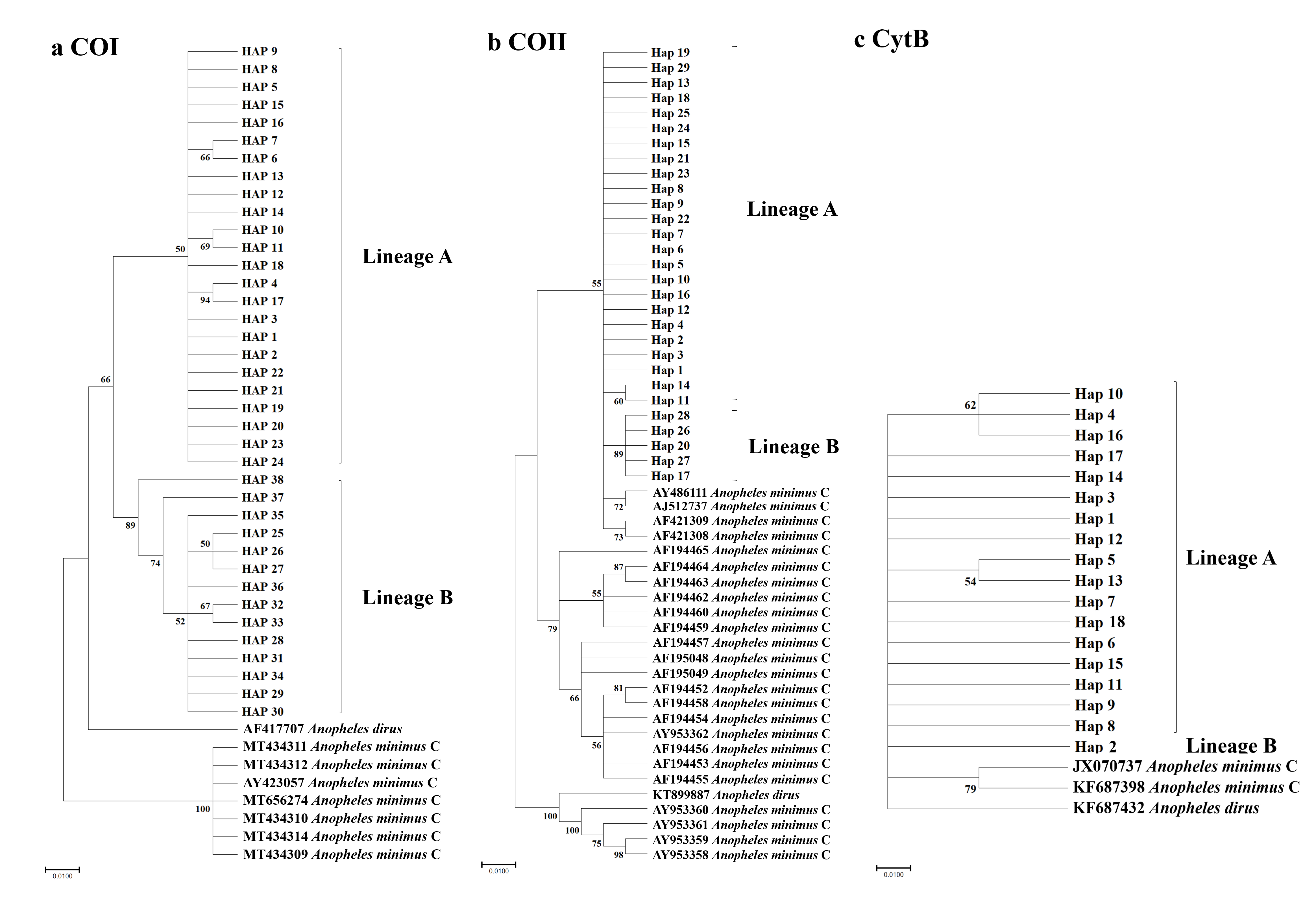

Supplement: Supplementary file 7 — Additional file 7: Figure S1. The maximum likelihood (ML) tree of two different genetical lineages of Anopheles minimus, the An. harrisoni as in the red circle and the An. dirus used as outgroup. Bootstrap values less than 50% were excluded in the phylogenetic tree. a The ML tree of the COI gene. b The ML tree of the COII gene. c The ML tree of the Cytb gene. The labels in the tree include haplotype codes, and color indicates the different populations. [file 13071_2021_4998_MOESM7_ESM.jpg]

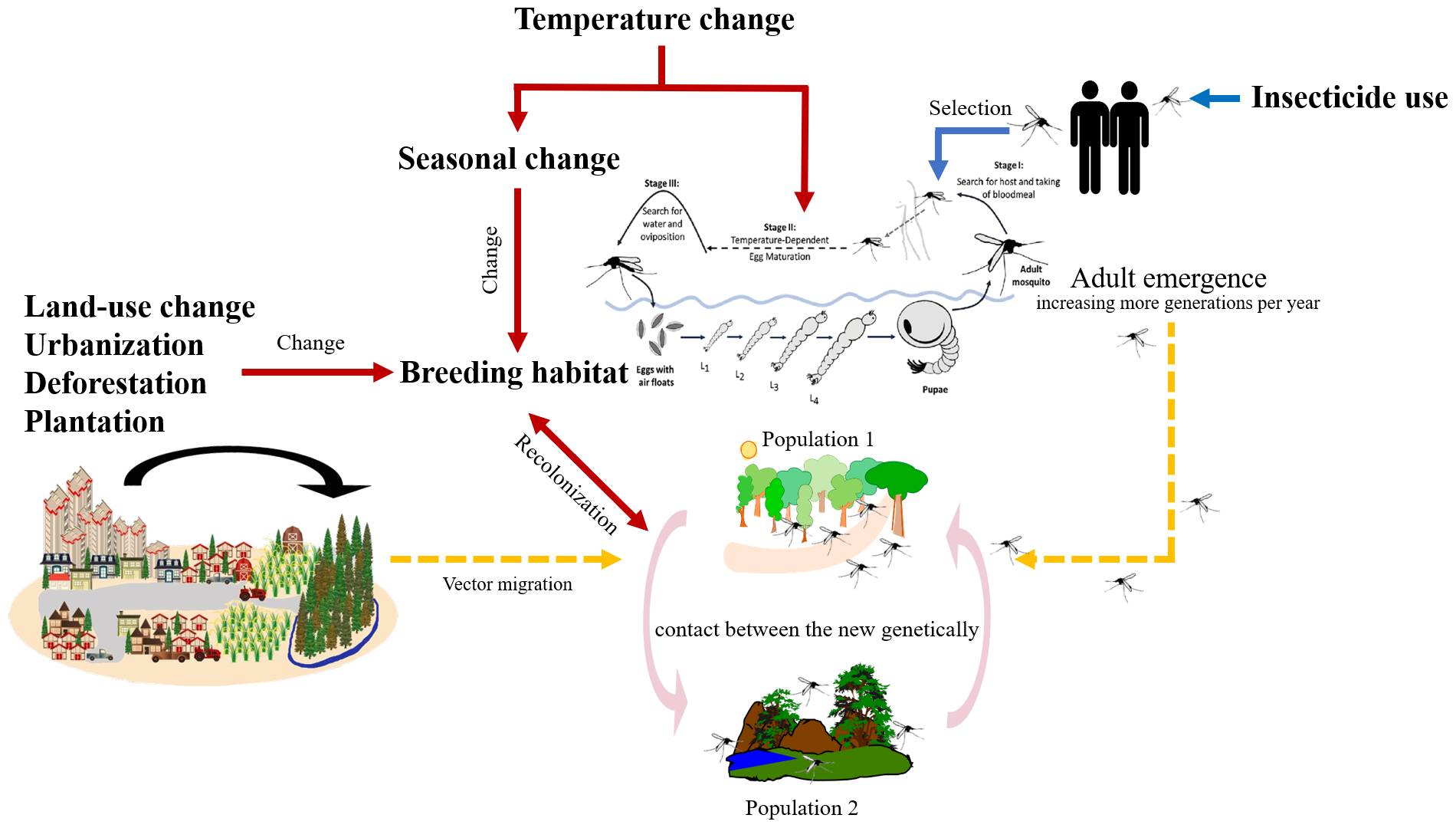

Supplement: Supplementary file 10 — Additional file 10: Figure S2. Fluctuations in environmental factors enhance gene flow, including temperature change, land-use change, urbanization, deforestation, plantation, and insecticide use. [file 13071_2021_4998_MOESM10_ESM.jpg]

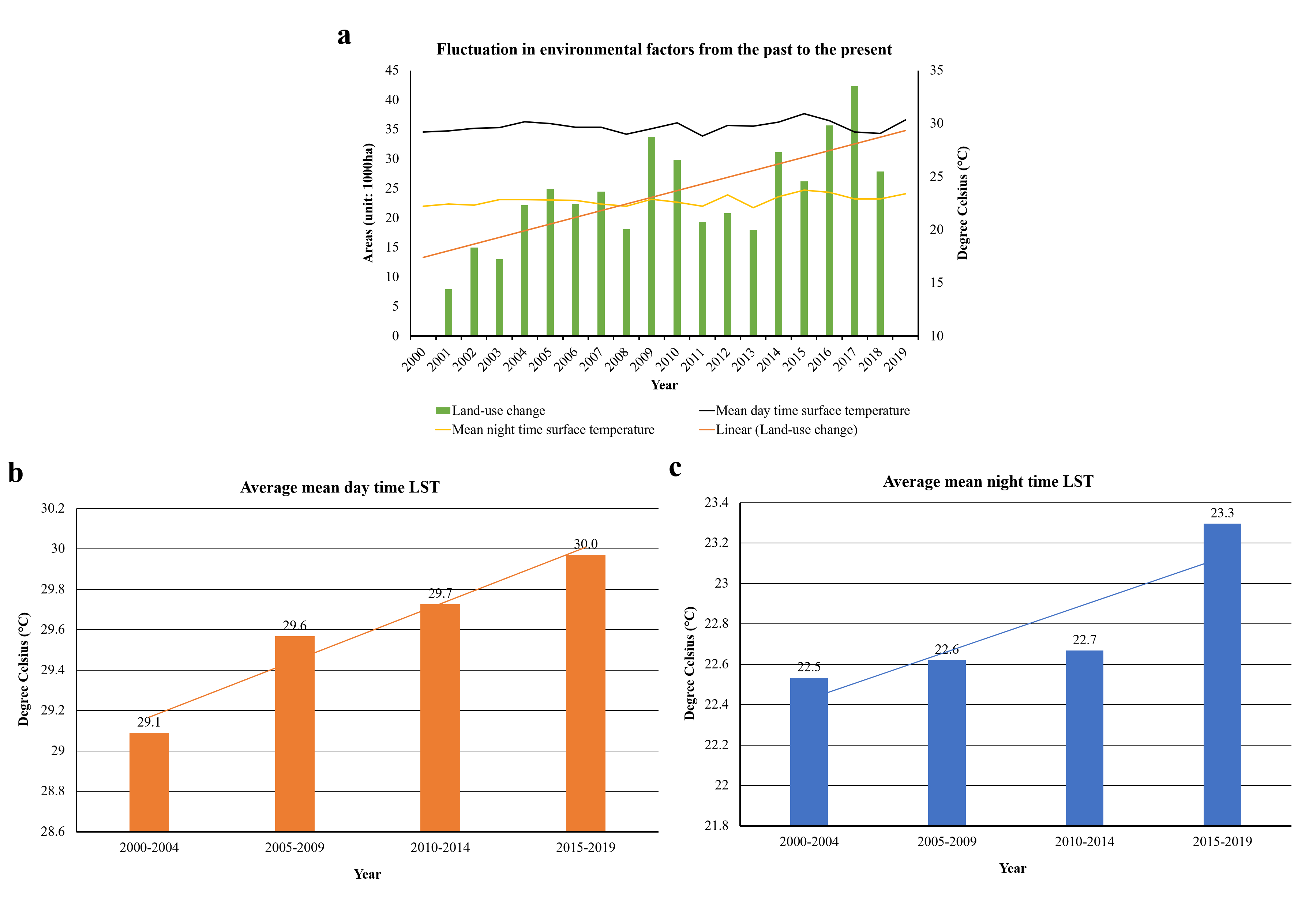

Supplement: Supplementary file 11 — Additional file 11: Figure S3. Fluctuation in environmental factors from the past to the present. a Fluctuation of environmental factors (land-use change, mean surface temperature by day and night). b The average of mean daytime temperature from 2000 to 2019. c Average mean nighttime temperature from 2000 to 2019. [file 13071_2021_4998_MOESM11_ESM.jpg]
