# Supplementary material for: Population genetic structure of the malaria vector Anopheles minimus in Thailand based on mitochondrial DNA markers
Source: Parasit Vectors. 2021 Sep 26;14:496. doi: 10.1186/s13071-021-04998-7 (PMC8474755; doi:10.1186/s13071-021-04998-7)
Supplement: Supplementary file 8 — Additional file 8: Table S7. Number of individuals, number of haplotypes, haplotype diversity (Hd), nucleotide diversity (π). [file 13071_2021_4998_MOESM8_ESM.docx]

**Additional file 8: Table S7.** Number of individuals, Number of haplotypes, Haplotype diversity (*Hd*), Nucleotide diversity (π)

| Population | | Tak | Surat Thani | Yala | Chanthaburi-Trat | Ubon Ratchathani | Overall |
| --- | --- | --- | --- | --- | --- | --- | --- |
| Lineage A | No. of individuals | 11 | 14 | 9 | 18 | 6 | 58 |
|  | No. of haplotype | 11 | 12 | 6 | 5 | 14 | 39 |
|  | *Hd* | 1.00000 | 0.97802 | 0.83333 | 0.95425 | 0.93333 | 0.97822 |
|  | π | 0.00766 | 0.00577 | 0.00262 | 0.00580 | 0.00365 | 0.00639 |
| Lineage B | No. of individuals | 4 | 6 | 5 | 6 | - | 21 |
|  | No. of haplotype | 4 | 4 | 5 | 5 | - | 16 |
|  | *Hd* | 1.00000 | 0.86667 | 1.00000 | 0.93333 | - | 0.96190 |
|  | π | 0.00317 | 0.00345 | 0.00243 | 0.00193 | - | 0.00295 |

*Abbreviations*: *Hd*, Haplotype diversity; π, nucleotide diversity
