# Supplementary material for: Population genetic structure of the malaria vector Anopheles minimus in Thailand based on mitochondrial DNA markers
Source: Parasit Vectors. 2021 Sep 26;14:496. doi: 10.1186/s13071-021-04998-7 (PMC8474755; doi:10.1186/s13071-021-04998-7)
Supplement: Supplementary file 9 — Additional file 9: Table S8.FST comparisons for all the five populations. [file 13071_2021_4998_MOESM9_ESM.docx]

**Additional file 9: Table S8.** *F_ST_* comparisons for all the five populations

| Population | | 1 | 2 | 3 | 4 | 5 |
| --- | --- | --- | --- | --- | --- | --- |
| 1 | Tak |  | 0.01113 | 0.14121 | 0.04355 | - |
| 2 | Surat Thani | 0.03083 |  | 0.12739 | 0.02154 | - |
| 3 | Yala | 0.36348 * | 0.20611 * |  | 0.26350 * | - |
| 4 | Chanthaburi-Trat | -0.0123 | 0.06326 | 0.41361 * |  | - |
| 5 | Ubon Ratchathani | 0.06657 | 0.17004 * | 0.58954 * | 0.09824 * |  |

**P* < 0.05
